# Associations of Adipocytokines with The Development of Cardiovascular Events in Young People

**DOI:** 10.3390/jpm13111582

**Published:** 2023-11-07

**Authors:** Alena D. Khudiakova, Yana V. Polonskaya, Victoria S. Shramko, Lilia V. Shcherbakova, Evgeniia V. Garbuzova, Elena V. Kashtanova, Yulia I. Ragino

**Affiliations:** Research Institute of Internal and Preventive Medicine—Branch of the Federal Research Center Institute of Cytology and Genetics, Siberian Branch of Russian Academy of Sciences (IIPM—Branch of the IC&G SB RAS), st. B.Bogatkova 175/1, 630089 Novosibirsk, Russia; yana-polonskaya@yandex.ru (Y.V.P.); nosova@211.ru (V.S.S.); 9584792@mail.ru (L.V.S.); stryukova.j@mail.ru (E.V.G.); elekastanova@yandex.ru (E.V.K.); ragino@mail.ru (Y.I.R.)

**Keywords:** adipocytokines, endpoints, cardiovascular events, young people

## Abstract

The research was aimed to study the associations of adipocytokines with the risk of cardiovascular events and to determine the threshold values of adipocytes for the prognosis of cardiovascular events in a young population. Materials and methods. The study is an epidemiological cohort study. The analysis included 1240 people aged 25–44 years. The endpoint was combined and included: death from cardiovascular disease, myocardial infarction, probable myocardial infarction, acute cerebrovascular accident, hospitalization for cardiovascular disease, and revascularization. Adipocytokines were determined with a MILLIPLEX panel. Results. In the examined population, 1.7% of cases of cardiovascular events were detected during cohort observation, of which 28.6% were fatal events. In men, cardiovascular endpoints were recorded 4.3 times more often than in women (17 (81%) vs. 4 (19%), *p* = 0.003). In individuals with cardiovascular events, arterial hypertension (2.6 times), diabetes mellitus (8.6 times), and overweight/obesity (1.5 times) were more often recorded compared to individuals without cardiovascular events. For tumor necrosis factor-alpha (TNFa), the threshold value was 2.5 pg/mL, with sensitivity assessment (Se) at 85.7% and specificity (Sp) at 83.3%. For amylin, the threshold value was 10.5 pg/mL, with Se at 73.7% and Sp at 67.0%. For pancreatic polypeptide (PP), the threshold value was 43.7 pg/mL, with Se at 85.7% and Sp at 56.7%. Conclusion. A method for assessing the risk of cardiovascular events in young people includes determining the levels of amylin, PP, and TNFa in blood serum. The cut-off points for predicting cardiovascular events were levels of amylin above 10.5 pg/mL, PP above 43.7 pg/mL, or a decrease in TNFa below 3.8 pg/mL.

## 1. Introduction

Despite the significant progress made in recent decades in the field of cardiovascular pathology, cardiovascular diseases (CVDs) remain the most urgent health problem in most countries in the world, including Russia. At the same time, the growth of CVD among young people is steadily increasing [1]. It is believed that one of the primary reasons for such data may be the late diagnosis and correction of risk factors for cardiovascular diseases, on the prevalence of which 60–75% of cardiovascular mortality depends [2]. Among the 250 currently known risk factors for cardiovascular diseases, they are unmodifiable (gender, age over 45 years in men and over 55 years in women, genetic factors, heredity—early onset of cardiovascular diseases in close relatives or sudden death in men <55 years, in women <65 years) and modifiable (lifestyle or behavioral factors, hypertension, dyslipidemia, impaired glucose tolerance, or type 2 diabetes mellitus) [3]. Given the high prevalence of abdominal obesity in the world [4], this factor is becoming one of the leading risk factors for the development of cardiovascular diseases.

Currently, much attention is paid to the concept of abdominal obesity causing a chronic systemic inflammatory reaction of mild severity, which occurs due to increased insulin resistance and increased production of inflammatory mediators due to an increase in the pool of adipocytes [5,6]. Adipokines are classified as proinflammatory and anti-inflammatory. It was found that in abdominal obesity, proinflammatory cytokines are increased due to anti-inflammatory cytokines, which contribute to a persistent, weakly expressed inflammatory response [7]. Visceral adipose tissue is considered a link between the formation of metabolic disorders and cardiovascular pathology. A number of studies demonstrate that adipokine imbalance is largely associated with increased risks of cardiometabolic diseases and their complications [8,9,10].

Data on the relationship between adipocytokine expression and age, gender, and other parameters are few and contradictory. In this connection, the identification of factors potentially influencing the course and prognosis of atherosclerosis-associated diseases continues to be an urgent problem requiring further study of the pathogenetic relationships of adipocytokines and risk factors for cardiovascular diseases, which will significantly improve the risk stratification for patients.

The study was aimed at evaluating the associations of adipocytokines with the risk of cardiovascular events and determining the threshold values of adipocytes for the prognosis of cardiovascular events in a young population.

## 2. Materials and Methods

For the purpose of creating a population sample, an area of Novosibirsk was chosen from the Territorial Compulsory Health Insurance Fund for Persons Aged 25 to 44. A sample of 2500 people was selected to reflect the population (with the help of random number generator). Young people are the most resistant to stimulation; thus, methods of gradual epidemiological stimulation were used, including mail invitations, phone calls, and media messaging. A total of 1512 participants were evaluated at the screening; the response rate was 60.5%. There were 1340 respondents to the poll, which was conducted from 2013 to 2017. After excluding from the study persons with fatal cases from causes unrelated to cardiovascular diseases (18 people), pregnant women, and women on maternity leave (82 people), 1240 people were included in the analysis, of which were 601 men (48.5%) and 639 women (51.5%). The average age of the examined was 36.98 ± 6.01 years.

The screening was conducted by a team of doctors trained in standardized epidemiological methods of screening examinations.

Waist circumference (WC) was measured with a centimeter tape, applying it horizontally in the middle between the lower edge of the costal arch and the sacral iliac bone.

Blood pressure was measured two times with an interval of 2 min on the right hand in a sitting position after a 5 min rest using an Omron M5-I automatic tonometer. We used the average value of two measurements. Arterial hypertension was recorded at systolic BP (SBP) ≥ 140 mmHg and/or diastolic BP (DBP) ≥ 90 mmHg.

The calculation of the body mass index (BMI) was carried out with the following formula: body weight (kg) divided by the square of height (m^2^).

The calculation of the glomerular filtration rate (GFR) was made in accordance with the national recommendations of KDIGO (Kidney Disease: Improving Global Outcomes) 2012 using the formula of CKD-EPI (Chronic Kidney Disease Epidemiology Collaboration). GFR ≥ 90 mL/min/1.73 m^2^ was considered optimal, and <90 mL/min/1.73 m^2^ was considered decreased [11].

Smokers were people who smoked at least one cigarette a day.

Blood sampling from the ulnar vein was performed 12 h after eating. Blood parameters such as lipid profile, glucose, and creatinine were measured with the enzymatic method using standard ThermoFisher (Waltham, Massachusetts, USA) reagents on an automatic biochemical analyzer, Konelab 30i (Finland). The conversion of serum glucose into plasma glucose was carried out using the formula plasma glucose (mmol/L) = −0.137 + 1.047 × serum glucose (mmol/L). Elevated blood levels of low-density lipoprotein cholesterol (LDL-C) were considered ≥116 mg/dL; elevated blood levels of non-high-density lipoprotein cholesterol (non-HDL-C) were considered ≥130 mg/dL; and elevated blood levels of triglycerides (TGs) were considered ≥150 mg/dL.

The levels of amylin, C-peptide, ghrelin, glucose-dependent insulinotropic polypeptide (GIP), glucagon-like peptide 1 (GLP-1), glucagon, interleukin 6, insulin, leptin, monocytic chemotactic factor 1 (MCP-1), and pancreatic polypeptide (PP) were determined with a multiplex analysis using the Human Metabolic Hormone V3 (MILLIPLEX) panel and tumor necrosis factor alpha (TNF-α). Human Adipokine Magnetic Bead Panel 1 was used to determine the levels of adiponectin, adipsin, lipocalin-2, plasminogen activator inhibitor type 1 (PAI-1), and resistin.

The endpoint was combined and included: death from cardiovascular disease, myocardial infarction, probable myocardial infarction, acute cerebrovascular accident, hospitalization for cardiovascular disease, and revascularization. Cases of cardiovascular diseases in the studied cohort were identified by comparing the “Acute Myocardial Infarction Register” (the WHO Program “Acute Myocardial Infarction Register” has been maintained in the IIPM Branch of IC&G SB RAS from 1982 to the present), medical documentation copied from the medical information analytical system, and the database of examined persons. The endpoints were evaluated for at least 5 years, starting in 2013.

SPSS software (version 13.0) was used to statistically process the results. The Kolmogorov–Smirnov criteria were used to verify that the distribution was normal. The data are presented for categorical variables as absolute and relative values (n%), and for continuous variables as Me [25; 75], where Me is the median and 25 and 75 are the first and third quartiles, respectively, due to the nonparametric distribution of the majority of the studied indicators. To compare two independent samples, the nonparametric Mann–Whitney U-test was employed. To compare the fractions, Pearson’s chi-squared criterion was employed. To assess the informativeness and resolution of the diagnostic test, a ROC analysis and sensitivity assessment (Se) were performed, which is defined as the proportion of patients who actually have cardiovascular events among those who tested positive (high risk), and specificity (Sp), which is defined as the proportion of people who do not have cardiovascular events among all those who tested negative (low risk). The criterion for choosing the optimal threshold for cutting off alipocytokine levels (the total number of points), which affects the ratio of sensitivity and specificity of the model, is the requirement of the maximum total Se and Sp. The critical significance level of the null hypothesis (p) was assumed to be 0.05.

The study was funded by the budget theme “Formation of cohorts of children, adolescents, and young people to study the mechanisms and features of the human life cycle in the Russian population” (No. 122031700115-7), with the support of the grant of the Russian Science Foundation No. 21-15-00022. This study was approved by the local Ethics Committee Protocol No. 167, dated 26 November 2019.

## 3. Results

**Figure 1:**
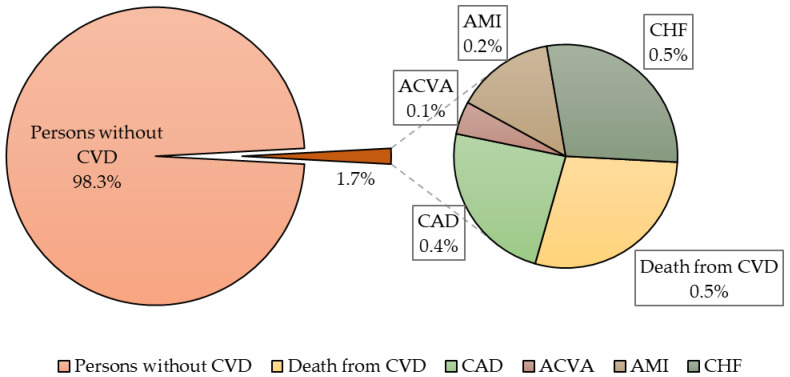
The prevalence of cardiovascular events in the population of 25–44 years of Novosibirsk. Note: CVD—cardiovascular disease, CAD—coronary artery disease, ACVA—acute cerebrovascular accident, AMI—acute myocardial infarction, CHF—chronic heart failure.

In the examined population, 21 (1.7%) cases of cardiovascular events were identified during cohort observation, of which 6 (28.6%) were fatal events (Figure 1). In men, cardiovascular endpoints were recorded 4.3 times more often than in women (17 (81%) vs. 4 (19%), *p* = 0.003).

Population characteristics depending on the onset of cardiovascular events are presented in Table 1. Individuals with cardiovascular events were 1.2 times older; had higher SBP scores by 1.1 times, DBP by 1.2 times, and BMI and waist circumference (WC) by 1.2 times; and had higher lipid profile scores (TG by 1.4 times, LDL-C by 1.3 times, HDL-C by 1.3 times, and TCH by 1.2 times) and fasted plasma glucose by 1.1 times compared to individuals without cardiovascular events, and did not differ in the levels of HDL-C and GFR CKD-EPI.

In addition, arterial hypertension (2.6 times), diabetes mellitus (8.6 times), and overweight/obesity (1.5 times) were more often recorded in people with cardiovascular events compared to people without cardiovascular events. There were no differences in the frequency of smoking and low physical activity (Table 2).

Individuals with cardiovascular events had 1.2 times higher PYY levels, 1.6 times higher C-peptide levels, 1.7 times higher PP levels, 1.8 times higher glucagon and IL-6 levels, and almost 2.5 times higher amylin levels; in turn, TNFα levels were 1.4 times lower compared with individuals without cardiovascular events (Table 3).

We used a single-factor logistic regression analysis to assess the risk of developing cardiovascular events in the young population of Novosibirsk. The analysis showed that with an increase in PP levels (by 1 pg/mL), the risk of cardiovascular events increased by 0.6%, and with an increase in amylin levels (by 1 pg/mg), by 3.3%. An inverse relationship was obtained for TNFα; with an increase in its level by 1 pg/mL, the risk of developing cardiovascular events decreased by 16.7%. (Table 4).

The model of the multivariate regression analysis confirmed the independent influence of the studied adipocytokines on the development of cardiovascular events. The logistic regression model included age, gender, diastolic blood pressure, low-density lipoprotein cholesterol, and body mass index. An increase in amylin levels, regardless of other factors, increased the risk of cardiovascular events by 3.6% and PP by 0.9%. An increase in TNFα levels reduced the risk of cardiovascular events by 19.2% (Table 5).

The determination of the cut-off values of amylin, PP, and TNFα for assessing the risk of cardiovascular events in young people was performed using the ROC analysis.

**Figure 2:**
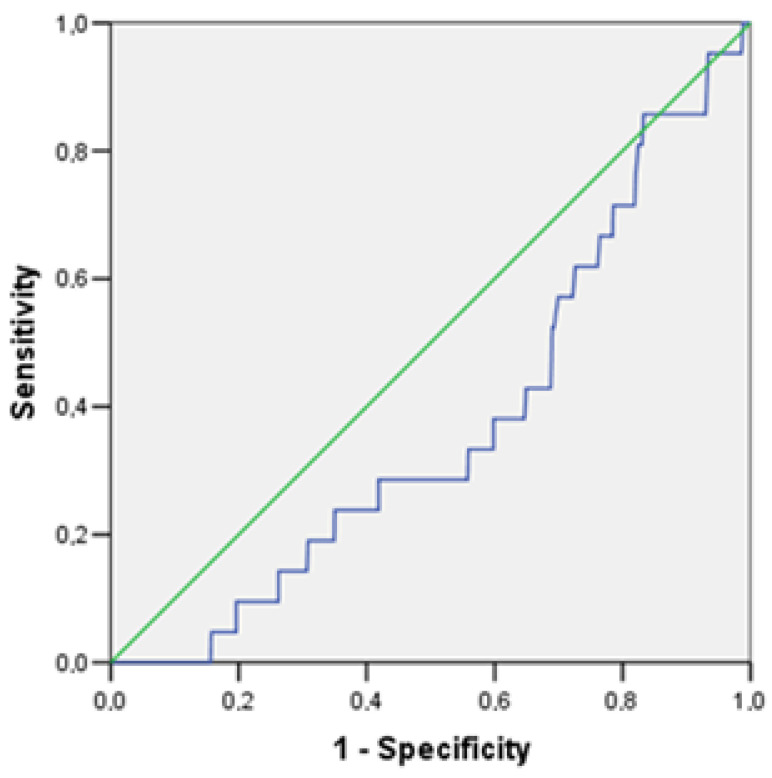
ROC curve of TNFa diagnostic ability for predicting the development of cardiovascular events in a young population of Novosibirsk.

For TNFα, the threshold value was 2.5 pg/mL, with Se at 85.7% and Sp at 83.3%. Taking into account the inverse relationship between the TNFα level and the risk of cardiovascular events, the quality of the diagnostic model can be considered average (the area under the curve is 0.373 (CI: 0.267–0.480; *p* = 0.046) (Figure 2).

**Figure 3:**
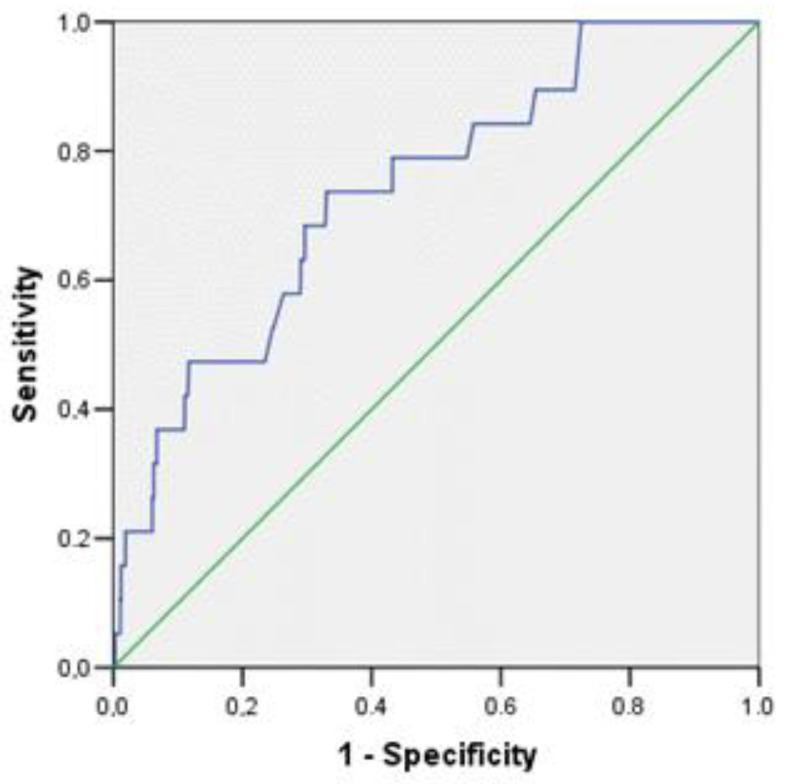
ROC curve of amylin diagnostic ability for predicting the development of cardiovascular events in a young population of Novosibirsk.

For amylin, the threshold value was 10.5 pg/mL. This indicator has an Se of 73.7% and an Sp of 67.0%; the quality of the diagnostic model turned out to be good (the area under the curve is 0.740 (CI: 0.630–0.849; *p* = 0.0003) (Figure 3).

**Figure 4:**
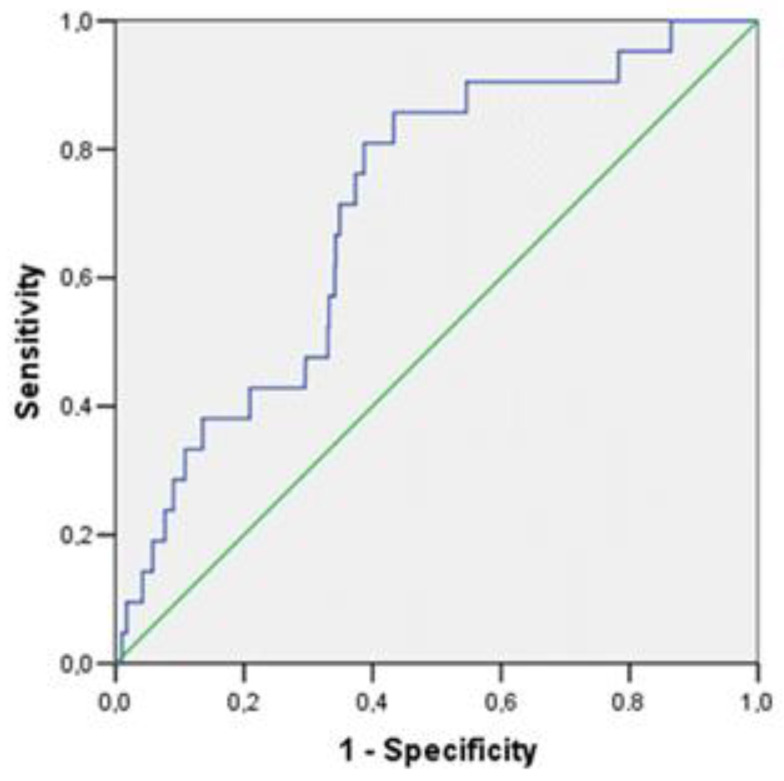
ROC curve of the diagnostic ability of PP for predicting the development of cardiovascular events in a young population of Novosibirsk.

For PP, the threshold value was 43.7 pg/mL. This indicator has an Se of 85.7% and an Sp of 56.7%; the quality of the diagnostic model is good (the area under the curve is 0.708 (CI: 0.608–0.807; *p* = 0.001) (Figure 4).

## 4. Discussion

The results obtained on the higher frequency of cardiovascular events in men aged 25–44 years compared with women correspond to the previously presented data [12,13], which most authors associate with the protective effect of endogenous estrogen on the cardiovascular system in women. In addition, gender also affects the range of risk factors, clinical symptoms, response to treatment, and prognosis of cardiovascular diseases [14]. When observing a young population, we found that men were almost 4.5 times more likely to have cardiovascular events compared to women.

The vast majority of epidemiological studies have concluded that the risk of cardiovascular diseases associated with obesity persists even after adjustment for concomitant risk factors [15], which makes obesity an independent predictor of the development of cardiovascular diseases. In addition to adverse hemodynamic effects and a variety of maladaptive changes in the structure and functions of the cardiovascular system [16], abdominal obesity can lead to the development of cardiovascular diseases by disrupting the balance of pro- and anti-inflammatory adipocytokines [17]. We also found that obesity, particularly abdominal obesity, along with a number of other cardiometabolic risk factors (arterial hypertension, diabetes mellitus, dyslipidemia) at baseline screening, was more often registered in individuals with developed cardiovascular events. It is worth noting that in the young population, we have not obtained differences in the frequency of such important risk factors as low physical activity and smoking. It is noteworthy that the risk of cardiovascular events was associated with some adipocytokines, regardless of BMI or the presence of abdominal obesity. This suggests that it is the involvement of systemic inflammation factors, as well as hormone-like substances produced by adipose tissue, that plays a key role in the development of cardiovascular events. At the same time, it should be noted that according to our data, two of the three adipokines associated with the risk of cardiovascular events (amylin and PP) are secreted by pancreatic cells and are hormones that control appetite [18,19].

TNFα increases the expression of many proinflammatory, procoagulant, proliferative, and proapoptotic genes in the vascular endothelium [20]. There is evidence that after a myocardial infarction, cardiomyocytes activated with ischemia and hypoxia synthesize a large amount of TNFα [21], which in turn can lead to relapses of cardiovascular events. Gonzálvez M. and co-authors proved that TNFα levels were not only higher in individuals who had an ischemic event than in those who did not, but also that in patients with ST-segment elevation myocardial infarction, TNFα levels in blood plasma 48 h after the onset of symptoms were independent predictors of cardiovascular events [22]. Similar results were obtained in the Paccalet A study with co-authors, which included 251 patients with ST-segment elevation myocardial infarction (mean age: 59 ± 12 years). In addition, the authors demonstrated that TNFα is a more powerful independent predictor of major adverse cardiovascular events compared to troponin I [23]. A large single-stage examination of 30,912 persons of European origin, which consisted of determining single-nucleotide polymorphisms associated with TNFα levels, showed that genetically predicted TNFα levels were positively associated with coronary heart disease (CHD) and ischemic stroke [24]. According to our data, an inverse relationship was obtained between the level of TNFα and the risk of cardiovascular events, which is not consistent with world data. It is believed that overexpression of TNFα and subsequent stimulation of the TNF receptor of type 1 cardiomyocytes cause contractile dysfunction, hypertrophy, fibrosis, and cell death, while a lower concentration of TNFα and subsequent stimulation of the TNF receptor of type 2 cardiomyocytes are protective. In addition to its concentration and receptor subtype, the effect of TNFα on the myocardium depends on the duration of its exposure and its localization. TNFα promotes protection against ischemic preconditioning, regardless of whether it is the first, second, or third protective window, and both TNF receptors are involved in the cascade of protective signal transmission [25]. This can probably explain the data we received. However, the place of TNFα in the pathogenesis of cardiovascular events certainly requires further study.

As seen with the increased activity of sympathetic nerves innervating adipose tissue and increased oxygen consumption, PP decreases food intake while simultaneously increasing energy consumption [18]. PP has a high affinity for the Y-type neuropeptide receptor Y4 and a lower affinity for the Y-type neuropeptide receptor Y1 and for the Y-type neuropeptide receptor Y5. Neuropeptide Y receptors are expressed in many places throughout the body and have an effect on vascular tone and tissue responses to ischemia [26]. Also, an increased fasting PP level is independently associated with vascular complications of diabetes mellitus and affects the retinal pathways, potentially affecting the survival of retinal neurons [27]. Also, several studies indicate no association between PP level and blood pressure in patients with autonomic dysfunction [28,29]. At the same time, there are no studies aimed at studying the associations of PP with cardiovascular diseases. We found that an increase in the PP level, regardless of other factors, increased the risk of cardiovascular events by 0.9%. Moreover, a cut-off point was determined for predicting cardiovascular events in young people.

Amylin plays an important role in glucose homeostasis, slowing gastric emptying, suppressing glucagon secretion, and exerting anorexigenic effects [19]. At the same time, an increase in the level of amylin leads to its accumulation in the myocardium and a change in Ca^2+^ fluxes in cell membranes [30]. It was found that amylin in the bloodstream reduces blood pressure by 35 mmHg for 30 min and has a rapid direct vasodilating effect. Tachycardia caused by an increase in amylin levels is caused by reflex activation of the sympathetic nervous system, secondary to hypotension [31]. In another study, it was found that the level of amylin in blood plasma increased with hypertension, and an increase in body mass index was associated with an increase in the level of circulating amylin. In addition, the authors concluded that the concentration of amylin in plasma is partially determined with heredity [32]. The peculiarities of the effect of amylin on the myocardium and vascular system make it a promising marker for determining cardiovascular risks; however, there are very few studies on the contribution of amylin to the development of cardiovascular diseases. In turn, we found that an increase in amylin levels in young people, regardless of other factors, increased the risk of cardiovascular events by 3.6%. For amylin, a cut-off point was also determined with the ROC analysis.

## 5. Conclusions

According to the data obtained, obesity (particularly abdominal obesity), arterial hypertension, diabetes mellitus, and dyslipidemia (including hyper-LDL-C, hyperTG, and hyper-non-HDL-C) were more often recorded in individuals with subsequent cardiovascular events. It is worth noting that in the young population, we have not obtained differences in the frequency of such important risk factors as low physical activity and smoking. This may indirectly indicate a greater contribution of the components of the metabolic syndrome to the development of cardiovascular diseases in young people.

Also, as a result of this study, adipocytokines were identified for the first time in which changes are predictors of the development of cardiovascular events in a young, able-bodied population. Among the 19 studied adipocytokines, substances were isolated to assess the risk of cardiovascular events in young people, including the determination of serum levels of amylin, pancreatic polypeptide (PP), and tumor necrosis factor alpha (TNFα). The cut-off points for predicting cardiovascular events determined levels for amylin above 10.5 pg/mL and for PP above 43.7 pg/mL, or for a decrease in TNFα below 3.8 pg/mL.

For the first time, cardiovascular outcomes over a 5-year period and associations of cardiovascular events with the concentration of adipocytokines were determined for young people living in the Siberian metropolis. The limitation of this study is the method of assessing endpoints, since it is impossible to assess cardiovascular outcomes in emigrated persons and persons who did not seek medical help.

## Figures and Tables

**Table 1:** Characteristics of the population of Novosibirsk at 25–44 years old.

Parameters	Individuals with Cardiovascular Events n = 21	Persons without Cardiovascular Events n = 1219	*p*
Age, years	43.5 [39.8; 45.0]	37.3 [31.8; 41.9]	0.0001
WC, cm	99.0 [88.3; 107.6]	86.0 [76.0; 96.0]	0.0001
SBP, mmHg	128.5 [122.3; 147.0]	120.0 [110.5; 130.0]	0.0001
DBP, mmHg	91.5 [82.5; 98.8]	78.5 [71.5; 87.0]	0.0001
BMI, kg/m^2^	29.0 [25.2; 31.6]	25.2 [22.1; 29.0]	0.002
TG, mmol/L	1.4 [0.9; 2.6]	1.0 [0.7; 1.4]	0.002
HDL-C, mmol/L	1.3 [1.1; 1.5]	1.1 [0.9; 1.5]	0.066
LDL-C, mmol/L	4.0 [2.9; 4.6]	3.2 [2.5; 3.7]	0.005
TCH, mmol/L	5.9 [4.8; 6.8]	5.0 [4.3; 5.7]	0.002
Non-HDL-C, mmol/L	4.8 [3.7; 5.4]	3.6 [3.0; 4.4]	0.001
GFR_CKD-EPI_, ml/min	103.1 [80.4; 106.9]	101.4 [90.4; 110.1]	0.424
Glucose, mmol/L	6.4 [5.6; 6.8]	5.7 [5.3; 6.0]	0.001

Note: WC—waist circumference, SBP—systolic blood pressure, DBP—diastolic blood pressure, TG—triglyceride, LDL-C—low-density lipoprotein, HDL-C—high-density lipoprotein, TCH—total cholesterol, Non-HDL-C—non-high-density lipoprotein cholesterol, GFR—glomerular filtration rate.

**Table 2:** Frequency of the main cardiometabolic risk factors depending on the presence of cardiovascular events.

Parameters	Individuals with Cardiovascular Events n = 21; 1.7%	Persons without Cardiovascular Events n = 1219; 98.3%	*p*
Physical activity less than 3 h/week (abs.; %)	13; 65.0%	812; 66.8%	0.863
Smoking (abs.; %)	8; 40.0%	413; 34%	0.572
AH (abs.; %)	11; 52.4%	244; 20.0%	0.0001
BMI ≥ 25 kg/m2 (abs.; %)	16; 76.2%	632; 51.9%	0.027
T2DM (abs.; %)	4; 19.0%	27; 2.2%	0.0001

Notes: AH—arterial hypertension, BMI—body mass index, T2DM—type 2 diabetes mellitus.

**Table 3:** Adipocytokine levels depending on the onset of cardiovascular events.

Parameters	Individuals with Cardiovascular Events n = 21	Persons without Cardiovascular Events n = 1219	*p*
	Me [25%; 75%]	Me [25%; 75%]	
Adiponectin, mcg/mL	34.0 [26.3; 139.3]	37.4 [25.7; 115.1]	0.372
Adipsin, mcg/mL	11.4 [9.4; 14.5]	11.7 [7.6; 14.1]	0.372
Lipocalin-2, ng/mL	493.0 [215.3; 1223.5]	379.2 [196.3; 1136.9]	0.694
Resistin, ng/mL	539.0 [176.8; 690.0]	170.9 [25.5; 598.0]	0.090
Amylin, pg/mL	14.2 [7.1; 22.7]	6.2 [3.4; 14.2]	0.0001
IL-6, pg/mL	2.4 [1.1; 6.5]	1.3 [0.6; 2.5]	0.025
PAI-1, ng/mL	29.4 [18.4; 41.3]	21.4 [13.0; 31.5]	0.106
C-peptide, ng/mL	1.3 [0.7; 2.0]	0.8 [0.3; 1.2]	0.013
Insulin, ng/mL	498.4 [327.8; 1172.2]	469.7 [296.0; 700.6]	0.261
Leptin, ng/mL	4846.2 [1922.1; 9370.0]	4382.7 [1689.3; 8271.9]	0.808
MCP-1, pg/mL	220.1 [118.6; 360.2]	238.3 [162.7; 319.6]	0.721
Ghrelin, pg/mL	66.2 [18.6; 111.1]	34.1 [18.6; 88.4]	0.289
TNFα, pg/mL	3.3 [2.6; 5.9]	4.7 [2.9; 7.3]	0.046
GIP, pg/mL	46.8 [18.3; 65.6]	26.1 [16.3; 51.4]	0.084
Glucagon, pg/mL	21.25 [11.5; 40.6]	11.5 [7.2; 20.9]	0.012
PP, pg/mL	65.9 [53.5; 121.3]	39.5 [22.8; 77.2]	0.001
GLP1, pg/mL	321.1 [174.9; 470.6]	283.8 [173.5; 492.7]	0.800
PYY, pg/mL	66.3 [60.3; 114.8]	56.1 [37.0; 74.3]	0.027
Secretin, pg/mL	17.7 [8.8; 26.8]	22.4 [16.0; 61.2]	0.212

Note: PAI-1—type 1 plasminogen activator inhibitor, IL–6—interleukin 6, MCP-1—monocyte chemoatractant protein–1, TNFα—tumor necrosis factor-alpha, GIP—glucose-dependent insulinotropic polypeptide, PP—pancreatic polypeptide, GLP-1—glucagon-like peptide-1, PYY—peptide tyrosine tyrosine, Me—median, 25% and 75%—1st and 3rd quartiles.

**Table 4:** The results of a logistic regression analysis of the association of adipokines with the risk of cardiovascular events with standardization by gender, age, HDL-C, DBP.

Parameters	OR	95% Confidence Interval (CI)		*p*
		Lower Bound	Upper Bound	
C-peptide, per 1 ng/mL	1.294	0.810	2.066	0.281
GIP, per 1 pg/mL	1.001	0.992	1.011	0.816
GLP1, per 1 pg/mL	0.999	0.997	1.001	0.186
PAI-1, per 1 ng/mL	1.019	0.990	1.050	0.201
IL-6, per 1 pg/mL	0.985	0.898	1.080	0.743
Insulin, per 1 ng/mL	1.000	1.000	1.000	0.997
Adipsin, per 1 mcg/mL	0.994	0.947	1.044	0.820
Lipocalin-2, per 1 ng/mL	1.000	0.999	1.001	0.744
Amylin, per 1 pg/mL	1.033	1.006	1.062	0.018
Ghrelin, per 1 pg/mL	1.001	0.998	1.003	0.677
Glucagon, per 1 pg/mL	1.010	0.992	1.029	0.255
Leptin, per 1 ng/mL	1.000	1.000	1.000	0.365
MCP-1, per 1 pg/mL	1.000	0.996	1.004	0.869
PP, per 1 pg/mL	1.006	1.000	1.013	0.056
PYY, per 1 pg/mL	1.006	0.995	1.017	0.283
Secretin, per 1 pg/mL	0.996	0.986	1.007	0.487
TNFα, per 1 pg/mL	0.833	0.699	0.992	0.040
Adiponectin, per 1 mcg/mL	1.000	0.993	1.006	0.918
Resistin, per 1 mcg/mL	1.000	0.999	1.001	0.943

Note: PAI-1—type 1 plasminogen activator inhibitor, IL–6—interleukin 6, MCP-1—monocyte chemoatractant protein–1, TNFa—tumor necrosis factor-alpha, GIP—glucose-dependent insulinotropic polypeptide, PP—pancreatic polypeptide, GLP-1—glucagon-like peptide-1, PYY—peptide tyrosine tyrosine, GFR—glomerular filtration rate, OR—odds ratio.

**Table 5:** Results of multivariate regression analysis of the association of adipokines with the risk of cardiovascular events.

Parameters	OR	95% Confidence Interval (CI)		*p*
		Lower Bound	Upper Bound	
Age, for 1 year	1.211	1.075	1.364	0.002
Gender, female vs. male	0.373	0.108	1.296	0.121
DBP, per 1 mm Hg	1.047	1.006	1.089	0.026
Non-HDL-C, per 1 mmol/L	1.365	0.939	1.985	0.103
BMI, ≥25 kg/m^2^ vs. <25 kg/m^2^	0.668	0.191	2.339	0.528
Amylin, per 1 pg/mL	1.036	1.003	1.069	0.032
PP, per 1 pg/mL	1.009	1.001	1.016	0.024
TNFα, per 1 pg/mL	0.808	0.669	0.975	0.026

Note: DBP—diastolic blood pressure, Non-HDL-C—non-high-density lipoprotein cholesterol, BMI—body mass index, TNFa—tumor necrosis factor-alpha, PP—pancreatic polypeptide, OR—odds ratio.

## Data Availability

Data is unavailable due to privacy.
